# Big Data in Insurance: Understanding Customer Resistance and Ethical Boundaries

**DOI:** 10.1007/s44206-026-00252-8

**Published:** 2026-03-12

**Authors:** Carmen Tanner, Nicole Witt, Urs Dahinden, Markus Christen

**Affiliations:** 1 Department of Finance, University of Zurich, Plattenstrasse 14, Zurich, 8032 Switzerland; 2 Department of Social Sciences, Zeppelin University, Am Seemooser Horn 20, 88045 Friedrichshafen, Germany; 3 University of Applied Sciences of the Grisons, Ringstrasse 34, Chur, 7000 Switzerland; 4 Department of Communication and Media Research, University of Zurich, Zürich, Switzerland; 5 Institute of Biomedical Ethics and History of Medicine, University of Zurich, Winterthurerstrasse 30, Zurich, 8006 Switzerland; 6 Digital Society Initiative, University of Zurich, Zürich, Switzerland

**Keywords:** Big data, Insurance, Contextual integrity, Customer resistance, Ethical values, Protected values

## Abstract

Big data is revolutionizing the insurance industry by enabling personalized risk assessments and pricing, yet these practices also raise ethical concerns. This study fills a gap in the literature by integrating the theories of contextual integrity and protected values to understand customer responses to the expanding use of big data applications in car insurance. These frameworks conceptualize ethical boundaries as limits to acceptable information flows and to trade-offs when data practices conflict with contextual norms or non-negotiable values. Using cross-national survey data from Switzerland (*N* = 1,080) and the United States (*N* = 1,084), we investigate customer objections to insurers collecting data on driving behavior, social media activity, online shopping, or creditworthiness. Our findings show that objections vary significantly by context, with collecting driving style data perceived as more acceptable than using credit information. While perceived benefits and trust in institutions lower objections, endorsement of protected values intensifies them. Customers holding strong protected values also exhibit absolute trade-off resistance, even when offered monetary incentives. A cluster analysis reveals four distinct customer segments, each demonstrating unique attitudes and resistance levels. Overall, the findings underscore the importance of acknowledging ethical boundaries and tailoring data strategies to heterogeneous customer segments.

**Supplementary Information:**

The online version contains supplementary material available at https://doi.org/10.1007/s44206-026-00252-8.

## Introduction

In the digital age, big data has become a vital asset for many industries, including insurance. By applying big data—encompassing the collection, processing, and analysis of vast and varied datasets (Mayer-Schönberger & Cukier, 2013)—insurers can assess individual risk profiles with greater precision and offer tailored products such as dynamic premium pricing (Barry & Charpentier, 2020; Kshetri, 2014). For example, car insurance providers can use telematics devices to monitor real-time driving habits, allowing them to correlate these behaviors with accident risks and adjust premiums accordingly. This practice is not limited to car insurance; life and health insurers are also increasingly using wearable devices to track health metrics and provide customized services.

Recent bibliometric analyses indicate a substantial rise in research on big data in insurance, particularly in the United States, alongside expectations of more granular risk assessment and product innovation (Eling et al., 2024; Ellili et al., 2023). At the same time, several scholars have noted that the shift toward fully individualized or behavior-based pricing has progressed more slowly and unevenly than initially anticipated (Charpentier & Vamparys, 2025; Francois & Voldoire, 2022). Studies also suggest that big data capabilities in the financial and service sectors are often used for marketing or service optimization rather than for personalized pricing. These debates highlight that the development of data-driven insurance products is evolving, with varying degrees of implementation across markets. Against this backdrop, it is increasingly important to understand how customers perceive both current practices and potential future applications of their data, particularly as insurers continue to experiment with data-intensive tools.

While these innovations promise significant benefits, they also raise ethical concerns, particularly regarding privacy. For instance, a recent report in the New York Times revealed that automakers were selling driving data to insurance companies without explicit customer consent, sparking public outrage (Hill, 2024). Such incidents underscore the discomfort customers feel when insurers access their personal data. Yet, while extensive research has examined consumer attitudes toward personal data collection in sectors like e-commerce (Gouthier et al., 2022; Kolotylo-Kulkarni et al., 2021), social media (Choi, 2023; Cloarec et al., 2024), or smart technologies (Schomakers et al., 2022), we still have a limited understanding of customers’ perceptions and responses to the use of big data in the insurance industry. Developing this understanding is crucial for insurers seeking to maintain trust and avoid reputational damage (Christen et al., 2019). Regulatory debates around data use and algorithmic decision-making also continue to evolve rapidly. A detailed legal analysis of these developments lies beyond the scope of this paper, which focuses on customer perceptions and ethical boundaries.

This research aims to address this gap by examining how insurance customers perceive and navigate the trade-offs between the benefits of big data and the associated ethical risks. Traditional frameworks, such as *privacy calculus theory* (Culnan & Armstrong, 1999; Dinev & Hart, 2006; Milne & Gordon, 1993; Xu et al., 2009) have been central to understanding these trade-offs but often oversimplify decision-making by treating personal information purely as an economic good. To provide a more nuanced perspective, we integrate insights from *contextual integrity* (Nissenbaum, 2010) and *protected values* (Baron & Spranca, 1997; Mullen & Skitka, 2006; Tetlock et al., 2000). These approaches emphasize that customers may have firm expectations regarding which types of personal information should not be shared with other parties, and which ethical principles should remain inviolate, regardless of monetary compensation. When customers believe these expectations are at risk of being violated, it can provoke strong reactions, including outrage (e.g., Ginges et al., 2007; Tetlock et al., 2000). We explore how these concepts apply to the context of car insurance, where big data-driven individualized risk profiles are part of public discourse and industry experimentation. According to Vrana and Singh (2023), the automotive industry (including production, consumption, and insurance) is a well-suited example for the analysis of digital transformation.

Through a cross-national survey with customers in Switzerland and the United States, we investigate how different big data applications intersect with customer perceptions and trade-off resistance. We believe this research makes several key contributions. First, it extends existing literature on data disclosure by examining customer reactions in an underexplored context: insurance. Second, it expands privacy calculus theory by incorporating the notion of trade-off boundaries, offering new insights into how customers navigate ethical concerns in personalized services. Third, it broadens the focus beyond privacy breaches to encompass a wider range of ethical issues. While privacy breaches are well-recognized as central concerns (Bansal et al., 2016; Rose et al., 2013; Sun et al., 2020), the evolving use of big data by insurers raises additional ethical challenges, such as the potential for unjust discrimination, violation of autonomy, or threats to the principle of solidarity (understood as shared risk) (Barry, 2020). To account for this, we will build on an exploratory study that identifies the ethical values customers perceive as compromised by insurers’ use of big data. Lastly, by acknowledging the heterogeneity among customers, this research identifies distinct segments with varying responses to big data practices in insurance.

## Theoretical Background

### Considering Benefits and Risks

In the insurance industry, the ability to access and analyze customer data has become essential for personalized risk assessments and customized products like tailored premiums. However, this growing reliance on data creates a critical trade-off for customers: the protection of personal data versus the potential benefits of customized services. Privacy calculus theory has been widely used to understand this trade-off. This framework posits that customers engage in a rational analysis, weighing the potential benefits of sharing their data against the perceived risks (Culnan & Armstrong, 1999; Dinev & Hart, 2006; Milne & Gordon, 1993; Xu et al., 2009). When the perceived benefits of sharing data outweigh the risks, customers are more willing to disclose their personal information.

The perceived benefits from data exchange can materialize in many ways. They may consist of a company offering reduced prices, new products, or additional services that provide added value. Personalization is also considered a key benefit for the customer, who may be willing to share personal data if they perceive a tangible reward. In this study, perceived benefits were used as a subjective indicator of how respondents evaluated the potential advantages of big data practices, rather than as a measure of actual benefits. Thus, we propose:

#### H1

Customers who perceive higher levels of benefits from big data will object less to its use for assessing individualized risk profiles.

In this study, we consider *lack of trust* in institutions as a proxy for perceived risks. According to prominent theories in organizational research, trust is defined as the willingness to accept vulnerability based on positive expectations of another party’s behavior (Mayer et al., 1995). Accepting vulnerability implies a willingness to take the risk of being harmed by the other party. In the insurance context, when customers allow insurers to collect and use their personal information, they inherently accept ethical risks, such as potential violations of privacy or inequitable treatment. Trust mitigates these concerns by providing customers with confidence that their data will be handled responsibly (Cook & Wall, 1980). Not surprisingly, trust has been identified as a critical factor for business success significantly influencing customer relationships, mitigating privacy concerns, and increasing the willingness of data sharing (Bansal et al., 2016; Gefen, 2000; Jayashankar et al., 2018; Pappas, 2016; Wu et al., 2012). So, we propose:

#### H2

Customers who express higher levels of trust will object less to the use of big data for assessing individualized risk profiles.

Surveys often highlight privacy as a major concern in the digital age (e.g., Bansal et al., 2016; Rose et al., 2013; Sun et al., 2020). Privacy is defined as the worry that the right to control personal information dissemination might be violated (Bansal et al., 2016; Goodwin, 1991). The so-called *privacy paradox*, however, suggests that customer behavior often contradicts their stated concerns. Despite claiming to value privacy, consumers frequently disclose personal data for minimal rewards (Bandara et al., 2017). This paradox has significant implications for firms, suggesting that monetary incentives might be used to overcome customer resistance to data collection. However, studies have yielded inconsistent results on this paradox (Gerber et al., 2018; Kokolakis, 2017). For example, a study by Lee et al. (2015) in the context of e-commerce shows that monetary rewards may even heighten privacy concerns, as they make customers suspicious of the motives behind data collection. Thus, monetary incentives for data disclosure can even backfire, intensifying privacy concerns rather than alleviating them.

The privacy calculus model, while widely used, has been criticized for assuming that consumers are rational and fully informed agents (Martin & Murphy, 2017). Decision-making is often influenced by cognitive biases, incomplete knowledge, and emotional factors (Acquisti et al., 2015; Kokolakis, 2017; Loewenstein & Lerner, 2003). These factors can lead to decisions that deviate from the rational model proposed by privacy calculus theory. Our research expands on this critique by exploring the boundaries of rational exchanges. We argue that privacy calculus theory does not fully account for situations where ethical concerns or contextual norms override rational cost-benefit analyses. These situations reflect value- and context-dependent boundaries to what customers perceive as acceptable data practices. By incorporating concepts of contextual integrity and protected values, we aim to offer a more nuanced understanding of why customers resist data collection, even when a rational analysis would predict otherwise.

### Contextual Integrity

Nissenbaum’s theory of contextual integrity (2004, 2010) offers a powerful lens for understanding why individuals might oppose the collection of personal information. The theory posits that data practices are evaluated based on norms of appropriateness and distribution, which vary depending on the context. These information flow norms dictate what types of information can be shared in a given context and how that information should be disseminated. For example, it might be acceptable to share medical information with a doctor but not with a marketing firm. Similarly, while it may be acceptable for a close friend to know certain personal details, it would be inappropriate for that information to be shared publicly without consent.

Contextual integrity is preserved when these norms are respected; compromising them can prompt objection and even moral indignation. These norms are deeply rooted in social and cultural expectations that evolve over time, creating a shared understanding of what constitutes acceptable and appropriate information flow within different contexts (Barkhuus, 2012; Martin, 2018; Nissenbaum, 2004). We contend that contextual integrity is also pivotal in understanding potential customer objection to big data practices in the insurance industry (Zimmer, 2018). For example, customers might find it appropriate for insurers to monitor driving habits to assess risk profiles, but they might object to the collection of data, such as social media activity or online shopping behavior. Such practices could be seen as overstepping boundaries, violating the customer’s contextual expectations.^1^ To explore these boundaries, we use a vignette-based approach in which respondents are presented with scenarios describing different big data applications used by car insurers to assess risk profiles. Each scenario describes an offer of the insurance that includes using personal data to determine individualized risk profile and adjust premiums about (a) the customer’s driving style, (b) the number of spelling errors in social media profiles, (c) the clothes bought in online shopping, or (d) the customer’s creditworthiness. By focusing on these specific settings, we aim to identify which types of big data practices are most likely to trigger customer objections due to perceived violations of contextual integrity. In line with earlier studies demonstrating the importance of context in data disclosure in social networks (Bilogrevic et al., 2013; Winter & Davidson, 2019), we posit:

#### H3

Different big data applications (that involve collecting different personal information) will affect the extent of customer objections.

### Protected Values

The concept of protected values offers a compelling framework for understanding customer objections to big data applications in the insurance industry. Traditional decision models assume that individuals weigh risks and benefits in a compensatory manner. Research on protected values, however, shows that individuals sometimes consider certain values or entities as inviolable, regardless of the material benefits involved (Baron & Spranca, 1997; Mullen & Skitka, 2006; Tanner & Medin, 2004; Tetlock et al., 2000). Examples include human life, justice, freedom, honesty, or nature. Such values are “not for sale” and form part of personal and collective identities (Fiske & Tetlock, 1997).

Research has consistently demonstrated that protected values are associated with non-compensatory decision patterns. Individuals endorsing protected values reject cost–benefit reasoning by revealing heightened resistance to trade-offs—either by setting extremely high thresholds for acceptability or by refusing any compromise, a phenomenon known as absolute trade-off resistance (Baron & Spranca, 1997). They also respond strongly when confronted with what they perceive as “taboo trade-offs,” often with anger or moral outrage (Baron & Spranca, 1997; Ginges et al., 2007; Tanner & Medin, 2004; Tetlock et al., 2000). Empirical work further shows that individuals who treat ethical principles such as honesty as non-negotiable are less susceptible to corruption (Tanner et al., 2022) and more likely to resist offers of financial gain that violate these values (Gibson et al., 2013). Taken together, these findings indicate that protected values can limit the applicability of cost–benefit model**s** because individuals may simply refuse to engage in trade-offs.

We propose that protected values are also relevant in the insurance context. Big data practices may be perceived as crossing ethical boundaries—not only by threatening privacy but also by undermining fairness or solidarity, which are central to insurance systems (Barry, 2020; Barry & Charpentier, 2020). For example, the use of unconventional data sources (e.g., shopping habits, credit records) may be seen as unjust or discriminatory, even when such practices offer tangible benefits. Customers who view privacy, equal treatment, or solidarity as protected values may therefore display the type of trade-off resistance described above: a reluctance to balance ethical concerns against potential benefits of data-driven services.

While our study does not test the underlying mechanism itself, we can build on a well-established body of prior research showing that protected values are reliably associated with non-compensatory decision patterns and trade-off refusal. This literature provides a strong theoretical basis for expecting that individuals who endorse protected values may react differently to big data practices. In line with this, we should observe in our study that customers endorsing protected values are, compared with other customers, more likely to object to big data practices and monetary compensation in exchange for personal data. Specifically, we hypothesize:

#### H4a

Customers who endorse protected values will object more strongly to the use of big data to determine individualized risk profiles.

#### H4b

Customers who endorse protected values will be more prone to exhibit absolute trade-off resistance, rejecting monetary compensation in exchange for personal data.

In our study, we focus on three values widely discussed in relation to big data and insurance: privacy, equal treatment, and solidarity. We acknowledge that these values are conceptually rich and sometimes contested in insurance debates (e.g., Baumann & Loi, 2023; Fleurbaey, 2008; Rudschies, 2023, for an overview, see Charpentier, 2024). These complexities cannot be fully captured in a survey-based design. Our aim is not to settle these normative positions, but to examine how lay customers respond to big data applications when presented with clearly defined and pre-tested descriptions of these values. To ensure shared understanding, the survey provided concise definitions of privacy, equal treatment, and solidarity (see Supplementary Information), which were developed and refined through pretesting. As shown in the exploratory study (see Appendix), these three values were also those most frequently identified by respondents as ethically salient in big-data-driven insurance scenarios. We thus use them as empirically grounded indicators of the broader ethical concerns customers associate with data-driven insurance practices. Specifically, privacy was defined in terms of control over personal information and protection from unwanted access; equal treatment emphasized non-discrimination in how individuals are assessed; and solidarity referred to mutual support within a collective (e.g., insurance pools).

### Segmentation

Segmentation has long been recognized as key to effective customer relationship management, particularly in marketing research. By identifying and addressing the specific needs and characteristics of distinct customer groups, firms may develop more effective communication strategies and tailor their offerings accordingly. In the context of big data use, recognizing customer heterogeneity must also be essential for insurers seeking to navigate the ethical complexities of data collection.

While previous research has segmented consumers based on privacy concerns toward data exchange and surveillance (Kumaraguru & Cranor, 2005; Pallant et al., 2022; Plangger & Montecchi, 2020), the insurance industry has, to date, traditionally relied on more readily available variables, such as demographics, claim history, and lifestyle factors, to segment customers (Wen et al., 2021). Though such variables provide some insight, they offer little insight into how customers perceive big data usage and the ethical concerns it raises.

Our research extends these traditional segmentation models by incorporating customers’ ethical beliefs and perceptions of big data practices. By understanding the ethical values that drive customer objections, insurers can better tailor their communication and data strategies to different customer segments to mitigate the risk of backlash (Morgan & Hunt, 1994). We pose the following research questions:

#### RQ1

Which customer segments can be identified based on perceived benefits, risks and protected values associated with the use of big data by insurances?

#### RQ2

How do those customer segments differ in terms of (a) their objections to big data applications, (b) their level of absolute trade-off resistance, and (c) demographic factors?

Although our study includes data from the US and Switzerland—and cultural, institutional, and regulatory contexts may play a role in how customers evaluate big data practices—a detailed theorization of such differences is not the central aim of this research. Our primary objective is to investigate general customer responses to big data applications in car insurance. Accordingly, cross-country and within-country patterns are considered in an exploratory manner rather than formulated as core hypotheses.

## Methodology

We conducted an exploratory study to identify which ethical values Swiss and US customers perceive as implicated in insurers’ use of big data. Respondents completed an online questionnaire that presented a scenario in which a car insurance company collects distinct types of personal data to assess individual accident risk and adjust premiums. Based on responses from 271 US and 168 Swiss respondents, we found that both groups recognized a broader spectrum of ethical values beyond privacy as pertinent to big data practices in the insurance industry (see Appendix).

Building on this, the main study focused on three key ethical principles: privacy, equal treatment, and solidarity. As Barry (2020) highlights, solidarity (understood as the sharing of risks) is a foundational ethical principle in insurance. Our primary goal was to assess how these values, when treated as protected, influence customer objections and trade-off resistance in response to big data practices. Data for the main study were collected via an online survey in autumn 2018, involving Swiss and US car insurance customers.

### Sample and Procedure

The survey targeted adults from the German- and French-speaking regions of Switzerland and from the United States, aged 18 years and older. Recruitment occurred through online panels provided by IPSOS, a global market and opinion research company, ensuring demographic quotas for gender, age, and education that mirror the Swiss and US adult populations. After data screening and cleaning for speeders, the final sample included 763 German-speaking, 317 French-speaking Swiss, and 1,084 English-speaking US respondents. Detailed sociodemographic characteristics are provided in Table 1.

**Table 1:** Characteristics of respondents

	Total (*n* = 2,164) (%)	US (*n* = 1,084) (%)	Switzerland German-speaking (*n* = 763) (%)	Switzerland French-speaking (*n* = 317) (%)
*Gender*				
Male	48.9	47.9	50.3	48.9
Female	51.0	51.9	49.7	50.8
Other / no answer	0.1	0.2	0.0	0.3
*A* *ge*				
< 31	17.1	19.8	14.5	13.6
31–40	16.2	19.4	12.7	13.9
41–50	14.9	10.9	19.0	18.6
51–60	22.0	19.4	24.8	24.0
> 60	29.9	30.5	29.0	30.0
*Residence*				
Big city	18.6	22.0	15.5	14.5
Suburbs	25.7	34.6	14.8	21.5
Town	31.1	30.1	29.8	37.5
Country village	19.2	4.5	37.6	24.9
Countryside	5.5	8.8	2.4	1.6
*Education*				
Low (less than high school)	7.9	8.7	6.9	7.9
Medium (high school or some college)	55.1	58.3	51.4	53.0
High (Bachelor or higher)	36.7	32.6	41.7	39.1
Other / no answer	0.2	0.5	0.0	0.0
*Employment*				
Employed	47.1	44.5	50.6	47.9
Self-employed	7.6	6.1	10.1	6.6
Student	3.8	3.3	3.7	5.7
Homemaker	7.0	9.2	4.3	5.7
Retired	18.9	14.4	23.5	23.7
Unemployed	6.4	8.4	3.8	6.0
Other / no answer	9.1	14.1	4.1	4.4

The survey was developed in German and translated into French and English using the translation-back-translation procedure (Brislin, 1976) to ensure linguistic accuracy. Pretesting ensured comprehensibility of the questions. The questionnaire included sections on ethical value concerns, digital services, and attitudes toward big data. Completion time was 10–15 min. 

### Measures

The independent and dependent variables were assessed as follows (for the exact item wording, see Supplementary Information).

To assess *perceived benefits of big data*, respondents were given a brief explanation of big data and asked to rate on a 5-point scale the extent to which they associate (dis)advantages with it (1 = *many disadvantages*, 5 = *many advantages*). *Lack of trust in institutions* to safeguard personal information, our proxy for perceived risk, was measured through four items assessing trust in government, media, business, and insurance on a scale from 1 (*distrust strongly)* to 5 (*trust strongly*). Endorsement of *protected values* was measured using three items for each value (privacy, equal treatment, and solidarity), adapted from Tanner et al. (2009). To ensure shared understanding, each value was accompanied by a succinct definition: privacy (control over and protection of personal information), equal treatment (non-discriminatory assessment of individuals), and solidarity (mutual support within a collective such as an insurance pool). These definitions were developed and refined and are reproduced in the Supplementary Information.

Participants were provided with brief definitions of the three ethical values to ensure shared understanding: privacy (control over and protection of personal information), equal treatment (non-discriminatory assessment of individuals), and solidarity (mutual support within a collective such as an insurance pool). These definitions were developed and refined through pretesting and are reproduced in the Supplementary Information.

Respondents indicated their agreement on a scale from 1 (*strongly agree*) to 5 (*strongly disagree*) with statements about whether the value in question was non-negotiable, should not be sacrificed, or could be considered in cost-benefit analyses (with the last item reversed).

To assess the dependent variable *objections to big data applications*, respondents were presented with vignettes illustrating how their insurance company might leverage big data for individualized risk assessment and premium adjustments. Four distinct offers were provided, developed in collaboration with experts from the insurance industry to ensure realism, each of them reflected a different big data practice.^2^ In each scenario, respondents were told that once a link between the data and the driver’s accident risk is established, premiums for low-risk drivers would be reduced, while those for high-risk drivers would remain unchanged.

(1) *Driving style*: This scenario described the insurer’s ability to access data on customer’s driving habits. Based on this recorded data, the insurer establishes a correlation between driving style and accident risk. (2) *Social media activity*: This scenario involved the collection of social media data. The insurance company extracts information on the number of spelling errors the driver makes in his or her social media profiles and links it to accident risk. (3) *Online shopping*: This scenario focused on the insurer using data from the driver’s online clothing purchases. Analyzing this data, the insurer infers accident risk based on gender, noting that female drivers generally pay less than males. (4) *Creditworthiness*: In this scenario, the car insurance company uses data of credit ratings. Analysis of this data reveals a connection between creditworthiness and accident risk, leading to wealthier individuals paying lower premiums. For each scenario, respondents rated their level of objection using two items: one measuring perceived outrageousness (1 = *not at all outrageous*, 5 = *highly outrageous*) and one measuring acceptability (1 = *not at all acceptable*, 5 = *highly acceptable*; this item was reversed for analysis). We acknowledge that some scenarios (e.g., the social-media spelling-error case) may appear less realistic; this variation is intentional, as creating critical variance in the contents allows us to test the theoretical mechanisms of contextual integrity and protected values.

The other dependent variable *trade-off resistance* was measured by asking respondents if they would accept monetary compensation in exchange for allowing their insurer to collect personal data (on driving style, social media activities, online shopping, and creditworthiness). Respondents chose among three options, which were subsequently dichotomized into categories representing resistance: 0 = *no resistance* (combining the options indicating acceptance in any case or under certain conditions), 1 = *absolute resistance* (refusal under any circumstances).

An exploratory factor analysis (EFA) was conducted on 21 items (excluding the single-item measures for perceived advantages of big data and trade-off resistance). We employed principal axis factoring with promax rotation to allow for correlations among factors (Fabrigar et al., 1999). Three items with factor loadings below 0.50 were removed. The final EFA, including 18 items, revealed five factors (eigenvalues > 1), accounting for 67% of the variance. Notably, protected values converged into a single factor, encompassing six items (across privacy, equal treatment, and solidarity). The four big data scenarios resulted in three distinct factors (driving style, creditworthiness, and a combined factor for social media and online shopping activities).

Reliability of the scales was confirmed through Cronbach’s alpha coefficients, all exceeded the recommended threshold of 0.70 (Robinson et al., 1991) (see Table 2). Discriminant validity was further supported via heterotrait-monotrait (HTMT) ratios, all below the recommended limit of 0.85, with a maximum HTMT ratio of 0.63 (Henseler et al., 2015).

**Table 2:** EFA factor loadings and reliability indices (Cronbach’s Alphas)

	Factor					Mean (SD)	Reliability (Alpha)
	1	2	3	4	5		
PV_equal1	**0.70**	0.07	-0.03	-0.01	0.01	4.11 (1.10)	0.86
PV_equal2	**0.74**	0.06	-0.02	-0.02	0.00	4.06 (1.12)	
PV_privacy1	**0.67**	0.09	-0.03	-0.09	-0.07	4.17 (1.03)	
PV_privacy2	**0.70**	0.10	-0.04	-0.08	-0.06	4.14 (1.08)	
PV_solidarity1	**0.69**	-0.14	0.03	0.10	0.03	3.74 (1.07)	
PV_solidarity2	**0.72**	-0.15	0.07	0.08	0.06	3.68 (1.08)	
Obj_shopping1	-0.04	**0.77**	-0.04	-0.04	-0.07	3.94 (1.20)	0.82
Obj_shopping2	0.04	**0.67**	0.02	0.06	0.07	3.68 (1.27)	
Obj_socmedia1	-0.01	**0.77**	0.03	-0.02	-0.02	3.93 (1.25)	
Obj_socmedia2	0.03	**0.67**	0.08	0.08	0.11	3.71 (1.29)	
Obj_driving1	-0.04	0.13	**0.73**	-0.08	-0.10	2.72 (1.27)	0.83
Obj_driving2	0.02	-0.04	**0.96**	0.03	0.05	2.48 (1.23)	
Obj_credit1	-0.02	0.24	-0.05	**0.57**	-0.12	4.00 (1.19)	0.83
Obj_credit2	0.00	0.01	-0.01	**0.99**	0.04	3.76 (1.24)	
Trust_government	-0.02	0.03	-0.02	0.06	**0.64**	2.77 (1.25)	0.77
Trust_media	0.01	-0.08	0.07	0.02	**0.55**	2.26 (1.14)	
Trust_business	-0.02	0.05	-0.01	-0.06	**0.83**	2.66 (1.07)	
Trust_insurance	0.00	0.06	-0.08	-0.04	**0.71**	2.88 (1.14)	

*Note*. Factors: 1 = Protected Values, 2 = Social Media/Online Shopping, 3 = Driving Style, 4 = Creditworthiness, 5 = Trust

To mitigate potential effects of common method bias, several procedural remedies were implemented: respondent anonymity was guaranteed, constructs were separated across different pages, and different scale formats were employed (Podsakoff et al., 2012). Additionally, we conducted Harman’s single factor test and factor analyzed all relevant variables (protected values, objections, trust, benefits of big data, trade-off resistance). The first factor accounted for only 28.3% of the total variance, which is well below 50%, indicating no significant common method bias.

## Results

### Comparison of Big Data Settings and Cross-Cultural Differences

To examine differences in customer objections to various big data practices (H3), we conducted an ANOVA with context, i.e., the type of big data application (driving style, social media/online shopping, and creditworthiness) as the within-subject factor, and country (US, Switzerland-German, Switzerland-French) as the between-subject factor (see Table 3).

**Table 3:** Descriptive statistics

	Objection Driving Style M (SD)	Objection Social Media/Online Shopping M (SD)	Objection Creditworthiness M (SD)	Trade-off Resistance
US	2.55 (1.11)	3.80 (0.99)	3.85 (0.99)	40.1%
Switzerland-German	2.57 (1.18)	3.76 (1.06)	3.97 (0.98)	51.0%
Switzerland-French	2.84 (1.23)	3.99 (0.97)	4.27 (0.89)	57.7%
Overall	2.60 (1.15)	3.81 (1.01)	3.96 (0.98)	46.5%

The analysis revealed a significant main effect of context (*F*(1.66, 3582.55) = 1252.57, *p* < .001, partial *η*² = 0.37), indicating that customer objections differed based on the type of big data being collected. Pairwise comparisons revealed that collecting driving style data elicited the fewest objections, while collecting creditworthiness data led to the highest level of objections (*ps* < 0.001). Additionally, there was a significant main effect of country (*F*(2, 2161) = 18.68, *p* < .001, partial η² = 0.02) with French-speaking Swiss expressing stronger objections compared to the other regions (*p*s < 0.001). French-speaking Swiss were also more resistant to trading personal data for monetary compensation (*X*²s > 4.09, *p*s < 0.05).

Furthermore, a significant interaction between context and country (*F*(3.32, 3582.55) = 3.15, *p* < .05, partial *η*² = 0.003) indicated that contextual influences varied across language regions: Unlike the Swiss, US respondents showed no significant difference in objections between the contexts of social media/online shopping and creditworthiness. 

### Predictors of Objection and Trade-Off Resistance

We tested H1, H2, and H4 using regression analyses to examine how perceived benefits of big data, trust in institutions, and protected values predict customer objections and trade-off resistance. Demographic variables (gender, age, education, place of residence, language region, and employment) served as control variables.

Table 4 depicts the results of the multiple regression analyses, with objections to the three big data practices—driving style (column 1), social media/online shopping activities (column 2), and creditworthiness (column 3)—as the criterion variables. Supporting H1 and H2, the β-coefficients show that higher perceived benefits and greater trust were associated with lower objections across all settings (*t*s < -2.89, *p*s < 0.01). Conversely, H4a was supported, as endorsing protected values was strongly linked to higher objections, particularly for the collection of social media/online shopping and creditworthiness data (*t*s > 7.98, *p*s < 0.001).

**Table 4:** Results of linear and logistic regression analyses

	Objection Driving Style	Objection Social Media/Online Shopping	Objection Creditworthiness	Trade-off Resistance
	β (S.E.)	β (S.E.)	β (S.E.)	OR
*Sociodemographics*				
Gender	0.03 (0.05)	0.04 (0.04)*	0.11 (0.04)***	1.31 **
Age	0.03 (0.00)	0.08 (0.00)***	0.04 (0.00)	1.01
Switzerland-German	0.01 (0.06)	-0.02 (0.05)	0.08 (0.05)***	1.65 ***
Switzerland-French	0.08 (0.07)***	0.04 (0.06)	0.13 (0.06)***	1.88 ***
Residence	-0.05 (0.02)*	0.03 (0.02)	0.04 (0.02)*	1.03
Education	-0.02 (0.04)	0.00 (0.04)	-0.04 (0.03)*	0.90
Employment	-0.03 (0.06)	0.00 (0.05)	-0.03 (0.04)	1.07
*Attitudinal Varia* *bles*				
Big data benefits	-0.10 (0.02)***	-0.15 (0.02)***	-0.10 (0.02)***	0.77 ***
Trust	-0.06 (0.03)**	-0.14 (0.02)***	-0.17 (0.02)***	0.64 ***
Protected values	-0.03 (0.03)	0.17 (0.03)***	0.20 (0.02)***	1.36 ***
* R*²	0.03	0.10	0.15	0.14
* F*	6.46***	25.40***	37.35***	

*Note. N* = 2114. Switzerland-German and Switzerland-French, Employment (1 = yes, 0 = no), and Gender (1 = female, 0 = male) were dummy-coded; high values for Residence represent a more rural area. The US was used as a reference for the country comparisons. For Trade-off Resistance, Nagelkerke R² is reported, otherwise the adjusted R²; OR = odds ratio

*p < .05, **p < .01, ***p < .001

The last column in Table 4 summarizes the results of the logistic regression, with trade-off resistance as a dichotomous dependent variable (1 = absolute resistance, i.e., no acceptance of monetary compensation in exchange for data collection). Consistent with our expectations (H4b), the odds of absolute trade-off resistance significantly increased among respondents endorsing higher levels of protected values (OR = 1.36, 95% CI [1.22 1.53], Wald *X*² = 28.15, *p* < .001). Conversely, the odds of resistance significantly decreased with greater perceived benefits of big data (OR = 0.77, 95% CI [0.71 0.84], Wald *X*² = 37.55, *p* < .001) and trust in institutions (OR = 0.64, 95% CI [0.57 0.71], Wald *X*² = 65.14, *p* < .001).

Additionally, while demographic variables played a minor role in our predictions, some cross-cultural differences were observed: compared to US respondents, German-speaking Swiss expressed more opposition to the creditworthiness setting, while French-speaking Swiss exhibited stronger objections to both the driving style and the creditworthiness setting (*ps* < 0.001).

### Segmentation

To address RQ1, we employed the Two-Step Cluster procedure in SPSS to identify customer segments based on perceived benefits of big data, trust, and protected values. Cluster analysis, as a data-driven approach, makes no prior assumptions about group membership and aims to identify subgroups by maximizing between-group separation while minimizing within-group variation (Hair et al., 2014). The Two-Step method combines a pre-clustering phase with hierarchical clustering, allowing for efficient handling of large datasets and mixed variable types (IBM, 2021). The optimal number of clusters was determined using Schwarz’s Bayesian Information Criterion (BIC), and clustering was based on a log-likelihood–based distance measure. A noise-handling threshold of 25% was applied to detect and remove outliers, resulting in the exclusion of five cases. All variables were standardized to z-scores prior to analysis. Although the BIC continued to decrease with additional clusters, improvements became marginal beyond four clusters. Therefore, a four-cluster solution was selected (BIC = 1221.47), balancing model fit and interpretability. This solution provided a nuanced view of customer attitudes toward big data applications. The overall quality of the clustering solution was assessed using the average silhouette measure of cohesion and separation, which yielded a fair value of 0.42. Additionally, we tested the stability of the four-cluster solution by repeating the procedure five times with different case orderings to assess sensitivity to different starting points. Comparable clusters emerged, and case assignments agreed by over 85% on average, indicating a robust and reproducible structure. Table 5 displays the sizes and profiles of each cluster.

**Table 5:** Cluster profiles

	Skeptical Protectionists	Pessimistic Protectionists	Trusting Protectionists	Pragmatists	
*Size of Cluster*	18.3%	32.8%	21.5%	27.4%	
*Main Variables*					*F*
Big data benefits^1^	0.62 ^a^	-1.16 ^b^	0.76 ^c^	0.38 ^d^	1444.05***
Trust^1^	-0.90 ^a^	-0.29 ^b^	1.06 ^c^	0.12 ^d^	518.90***
Protected Values^1^	0.61 ^a^	0.33 ^b^	0.52 ^a^	-1.13 ^c^	786.46***
*Behavioral Variables*					
Obj_driving^1^	0.00 ^ab^	0.10 ^a^	-0.18 ^b^	0.00 ^a^	6.91***
Obj_soc media & online shop^1^	0.17 ^a^	0.29 ^a^	-0.17 ^b^	-0.29 ^b^	46.81***
Obj_creditworth^1^	0.26 ^a^	0.27 ^a^	-0.19 ^b^	-0.31 ^b^	53.77***
					*X*²
Trade-off resistance	50.4% ^a^	59.1% ^b^	35.8% ^c^	36.7% ^c^	89.96***
*Sociodemographics*					
*Gender*					
Male	45.0% ^a^	46.9% ^a^	51.5% ^a^	53.4% ^a^	9.42*
Female	55.0% ^a^	53.1% ^a^	48.5% ^a^	46.6% ^a^	
*Age*					
< 31 years	15.8% ^ab^	13.7% ^a^	21.8% ^b^	18.2% ^ab^	28.13**
31–40 years	19.6% ^a^	14.4% ^a^	16.5% ^a^	15.7% ^a^	
41–50 years	15.0% ^a^	14.3% ^a^	13.2% ^a^	16.7% ^a^	
51–60 years	22.0% ^ab^	24.9% ^a^	17.6% ^b^	21.2% ^ab^	
> 60 years	27.6% ^a^	32.7% ^a^	31.0% ^a^	28.2% ^a^	
*Country*					
US	62.0% ^a^	44.2% ^b^	56.3% ^a^	44.1% ^b^	74.95***
Switzerland-German	23.0% ^a^	38.9% ^b^	27.9% ^a^	45.3% ^b^	
Switzerland-French	15.0% ^ab^	16.9% ^a^	15.8% ^ab^	10.7% ^b^	
*Residence*					
Big city	17.1% ^ab^	16.1% ^a^	23.5% ^b^	18.6% ^ab^	31.55**
Suburbs	26.6% ^a^	24.6% ^a^	24.2% ^a^	27.9% ^a^	
Town	34.6% ^a^	31.6% ^a^	32.1% ^a^	27.5% ^a^	
Country village	15.5% ^ab^	22.0% ^a^	14.3% ^b^	22.0% ^a^	
Countryside	6.2% ^a^	5.6% ^a^	5.9% ^a^	4.0% ^a^	
*Education*					
Low (less than high school)	11.2% ^a^	6.6% ^ab^	5.7% ^b^	7.9% ^ab^	12.31
Medium (high school or some college)	55.3% ^a^	53.9% ^a^	57.0% ^a^	54.5% ^a^	
High (Bachelor or higher)	33.5% ^a^	39.5% ^a^	37.3% ^a^	37.6% ^a^	
*Employment*					
Employed	40.1% ^a^	44.5% ^ab^	52.3% ^b^	51.6% ^b^	35.18**
Self-employed	8.8% ^a^	9.1% ^a^	6.4% ^a^	6.5% ^a^	
Student	5.2% ^a^	3.7% ^a^	3.7% ^a^	2.8% ^a^	
Homemaker	9.6% ^a^	6.1% ^a^	5.7% ^a^	6.9% ^a^	
Retired	17.3% ^a^	21.5% ^a^	16.9% ^a^	19.4% ^a^	
Unemployed	7.5% ^a^	5.6% ^a^	6.8% ^a^	5.3% ^a^	
Other / no answer	11.6% ^a^	9.5% ^a^	8.1% ^a^	7.4% ^a^	

*Note. N* = 2117. Values within each row that have different superscripts differ significantly after Bonferroni adjustment (*p* < .05). ^1^ z-score values

*p < .05, **p < .01, ***p < .001

To address RQ2, we conducted ANOVAs and *X*²-tests to assess overall differences between the clusters. Pairwise comparisons with Bonferroni correction were also performed (see Table 5). Not surprisingly, the clusters differed significantly across all three variables used in the cluster analysis (*F*s > 518.90, *p*s < 0.001). More importantly, the clusters also displayed significant differences in objection levels (also standardized to z-scores for ease of interpretation; *F*s > 6.91, *p*s < 0.001) and trade-off resistance (*X*²(3) = 89.96, *p* < .001), with notable variations in demographic factors like gender, age and country (*X*²s > 9.42, *p*s < 0.05).

#### Cluster Characteristics

We characterize the four clusters of customers according to their concern for protected values (as a proxy for ethical concerns), perceived advantages of big data (as a proxy for perceived benefits), and (lack of) trust in institutions (as a proxy for perceived risks).

We labeled the first segment *Skeptical Protectionists* (18.3%). This group recognizes the potential benefits of big data, suggesting a general acceptance of data collection if clear advantages are evident. However, their strong endorsement of protected values indicates a firm stance against compromising core ethical values. Low trust in institutions further makes them skeptical about the security of their personal data. Consequently, members of this group are expected to object to data collection practices and exhibit trade-off resistance, even while acknowledging the potential benefits of big data. As can be seen in Table 5, their objections are above-average, particularly concerning social media/online shopping data and creditworthiness. Furthermore, about 50% of this group displays absolute resistance to exchanging their data for monetary compensation. It is interesting that the other roughly 50% is willing to exchange data for monetary rewards, demonstrating the privacy paradox. Demographically, this cluster includes the highest proportion of US citizens. 

We labeled the second cluster *Pessimistic Protectionists* (32.8%). Like Skeptical Protectionists, this group holds strong protected values and lacks trust in data protection. However, what distinguishes them from the Skeptical Protectionists is their markedly negative view of big data, perceiving no benefits from its use—a perspective that is distinctly more pessimistic in nature. Consequently, they exhibit even stronger objections to data collection and are more reluctant to share personal data with insurers. As shown in Table 5, this group reveals above-average objection rates and elevated trade-off resistance, with 59% rejecting monetary compensation for their data. Notably, they also object to the collection of driving style data, which is more widely accepted by other segments. This cluster includes a relatively high proportion of Swiss customers (both German- and French-speaking) and older individuals.

We labeled the third segment *Trusting Protectionists* (21.5%). Despite endorsing protected values, this group exhibits high trust in institutions and optimism about the benefits of big data. These customers are expected to be generally open to data collection and trade-offs, balancing ethical concerns against perceived benefits. As shown in Table 5, these attitudes are reflected in their minimal objections to data collection and negligible trade-off resistance. Demographically, this segment has the highest proportion of younger individuals (under 31) and a relatively high proportion of US citizens.

We labeled the fourth segment *Pragmatists* (27.4%). Ethical values are not a concern for this group, as indicated by their negative score for protected values. They see some positive value in big data and demonstrate moderate trust in institutions. Viewing data as a tradable commodity, this group shows minimal objection to data collection and little trade-off resistance (see Table 5). Pragmatists are practical in their approach to data use, prioritizing tangible benefits over ethical considerations. This cluster includes a relatively high proportion of Swiss Germans and males.

## Discussion

This study offers valuable insights into customer responses to big data practices in the car insurance industry. We discuss the theoretical contributions, practical implications, limitations, and offer suggestions for future research.

### Theoretical Contributions

This study advances understanding of customer resistance to big data in insurance by extending privacy calculus theory (Culnan & Armstrong, 1999; Dinev & Hart, 2006; Milne & Gordon, 1993; Xu et al., 2009). While confirming that perceived benefits and trust in institutions reduce objections (H1 and H2), our findings show that contextual integrity and protected values impose clear limits on compensatory reasoning (H3 and H4). By integrating these perspectives, the study explains both context-dependent objections and non-compensatory resistance, highlighting where traditional privacy-calculus models reach their limits.

Consistent with contextual integrity theory (Nissenbaum, 2004, 2010), customer objections vary systematically by data type, confirming H3. Using data on social media or creditworthiness to assess risk profiles seem to violate contextual expectations, provoking heightened objections (especially among French-speaking Swiss respondents), whereas collecting driving behavior data is perceived as more acceptable—we suggest, because it directly relates to driving risks. These findings highlight the importance for insurers to consider the contextual expectations which outline clear boundaries for what customers perceive as acceptable uses of personal data, helping to avoid negative reactions and erosion of trust when implementing big data practices (Martin, 2018).

Protected values (Baron & Spranca, 1997; Tetlock et al., 2000) offer another critical dimension to privacy calculus theory by emphasizing non-negotiable ethical principles that establish limits to acceptable trade-offs. Customers endorsing protected values are less likely to engage in trade-offs, even when offered financial benefits. Our findings support H4a and H4b, showing that these customers exhibit absolute trade-off resistance, rejecting compensation for personal data. This challenges the assumption that economic incentives can always mitigate objections. Both contextual integrity and protected values emphasize that personal data cannot be viewed solely as an economic good, and integrating these concepts enriches privacy calculus theory by offering a more comprehensive understanding of customer objections and resistance to big data.

### Practical Implications

The findings carry important implications for insurers increasingly relying on big data for personalization and pricing. A key takeaway is the need to respect trade-off boundaries and contextual norms. Aligning data strategies with customers’ contextual expectations and protected values may help insurers mitigate resistance and build trust. This involves collecting data that customers perceive as relevant to the insured risks, such as driving behavior, while avoiding data seen as unrelated—such as social media activities—which tend to provoke backlash and erode trust (Martin, 2018). Consistent with prior research, respecting normative expectations around data use may function as an informal ethical contract that supports long-term trust (Poppo & Zenger, 2002; Pirson et al., 2015). In this sense, insurers who consistently demonstrate ethical commitments in their data practices—e.g., through regular audits and certifications—may be better positioned to sustain trust.

Another takeaway is that financial incentives are not universally effective. For customers strongly endorsing protected values, attempts to trade ethical concerns for monetary benefits may provoke resistance or outrage (Ginges et al., 2007; Tetlock et al., 2000). Our findings highlight the risks associated with incentivizing data sharing among these customers, as some may view these offers as commodifying their ethical principles, leading to increased resistance. Thus, attempts to trade off protected values for economic gain can be counterproductive.

In our study, resistance to monetary offers in exchange for personal data was especially strong among Skeptical and Pessimistic Protectionists. However, even among these groups, some were willing to consider the benefits against the risks. For instance, about half of the Skeptical Protectionists were open to trading their data for financial rewards, reflecting the privacy paradox. In the past, the observation that protected values can be malleable has fueled the suspicion that such values may serve as posturing or impression management (Baron & Leshner, 2000). However, other studies have found that individuals endorsing protected values often experience emotional distress when compromising them (Hanselmann & Tanner, 2008; Lichtenstein et al., 2007), suggesting that these values may nonetheless be genuine, even when customers are occasionally willing to make trade-offs.

Moreover, the cluster analysis highlights substantial heterogeneity among customers, indicating that uniform communication strategies are unlikely to succeed. The resulting segmentation offers a theoretically grounded classification of response patterns that has not previously been examined in the insurance context. For example, while Trusting Protectionists may respond positively to assurances about ethical standards and governance, Pragmatists appear more receptive to benefit-focused messaging. Furthermore, distinguishing between Skeptical and Pessimistic Protectionists proves valuable, as these two groups–despite sharing low institutional trust and strong protected values–differ markedly in their openness toward big data. Skeptical Protectionists, who remain open to potential benefits but are concerned about ethical implications and lack institutional trust, may be reassured through transparency and ethical safeguards, whereas Pessimistic Protectionists, who are highly resistant to data collection, may require opt-out options that preserve traditional insurance models without participating in big data-driven personalization.

### Limitations and Future Work

Like any study, ours has limitations. One limitation concerns the use of two single-item indicators—perceived benefits of big data and trade-off resistance. While single-item measures can be appropriate for clear and unidimensional constructs and help reduce respondent burden in complex surveys (Wanous et al., 1997; Gardner & Cummings, 1988), we acknowledge that multi-item scales generally offer higher reliability. In our study, these items served as pragmatic proxies rather than core theoretical constructs, and the overall pattern of results is consistent with theoretical expectations. Nevertheless, future research would benefit from using validated multi-item instruments to capture these constructs in a more nuanced way.

Additionally, our explorative study has highlighted that customers consider a broader range of ethical values beyond privacy when evaluating big data practices of insurances. For simplicity and coherence in factor analysis, we combined customers’ commitments to privacy, equal treatment, and solidarity into one protected value scale. These values, however, represent distinct ethical concerns. Future research could delve deeper into the unique impact of each value on customer behavior toward big data practices.

Another limitation is the reliance on self-reported data, which may be influenced by social desirability bias. Although vignette-based judgments cannot fully reproduce actual behavioral dynamics, they are an established method in ethical decision-making research and typically yield insights that relate meaningfully to real-world behavior, especially when scenarios are context-rich (FeldmanHall et al., 2012; Webb & Sheeran, 2006). Although the data were collected in 2018, they offer a valuable baseline for how customers drew ethical and contextual boundaries around big-data practices before recent regulatory and AI-related debates. Mapping these early boundaries remains important for understanding how expectations evolve over time. Future research could consider incorporating behavioral data to confirm our findings. Longitudinal research would also be valuable in examining how customer attitudes evolve as digital technologies and data practices continue to develop.

Lastly, while demographic and cultural factors were not the primary focus of this study, some notable cross-regional differences emerged that warrant further investigation. In particular, French-speaking Swiss respondents showed higher levels of objection and trade-off resistance compared to German-speaking Swiss and US respondents. Existing research acknowledges that such intra-country differences are plausible but not well understood (Thir et al., 2023). Future research should therefore examine both cross-country and intra-country cultural dimensions of data ethics more systematically, especially in a globalized market where insurers operate across diverse cultural contexts.

## Supplementary Information

Below is the link to the electronic supplementary material.

Supplementary Material 1

## Data Availability

The data of this study are available from the authors upon reasonable request.

## Footnotes

1 We acknowledge that violations of contextual integrity constitute only one mechanism relevant for understanding moral judgments in the insurance sector. Additional factors include the causal interpretations and moral categorizations that shape fairness perceptions of insurance markets (Kiviat, 2019), as well as broader considerations related to trust in AI applications in general (Afroogh et al., 2024) and in the insurance domain specifically (Noordhoek, 2023).

2 Scenario realism was validated with practitioners from a major Swiss insurance company: eleven experts reviewed the draft scenarios, and their feedback was consolidated in a workshop with five insurance experts and the research team.
