# Supplementary material for: Big Data in Insurance: Understanding Customer Resistance and Ethical Boundaries
Source: Digit Soc. 2026 Mar 12;5(1):9. doi: 10.1007/s44206-026-00252-8 (PMC13675310; doi:10.1007/s44206-026-00252-8)
Supplement: Supplementary file 1 [file 44206_2026_252_MOESM1_ESM.docx]

**Appendix —** *Exploratory study*

Prior to our main survey, we designed an exploratory study to identify which ethical values customers see as being implicated in the use of big data applications by insurance companies. We solicited US respondents via Amazon Mechanical Turk (MTurk) and Swiss respondents via an openly accessible survey link within a large Swiss university. After cleaning the data of those respondents who exhibited excessively rapid completion times or failed attention checks, the final dataset comprised 271 US and 168 Swiss respondents.

Through a questionnaire, these respondents were presented with a hypothetical scenario depicting the use of big data in determining car insurance premiums. This scenario outlined a situation in which a car insurance company gathers diverse types of information about their customers, including data on driving behavior (monitored via a black box installed in the car), data gathered from the driver’s social networks (e.g., Facebook), and data regarding the driver’s creditworthiness.

The respondents were informed that the insurance company would use this data to assess correlations between various factors and the driver’s propensity for accidents. Consequently, drivers might face elevated premiums if their assessed accident risk surpasses that of other customers. After this description, the respondents were presented with a list of ethical values—privacy, equal treatment, solidarity, transparency, security, autonomy, and freedom—each accompanied by a succinct definition (see Supplementary Information for the detailed definitions). For each value, the respondents indicated whether they perceived the corresponding ethical principle to be implicated (scored as 1) or not implicated (scored as 0) in the described scenario.

As summarized in Table A1, the respondents identified a broader spectrum of ethical values beyond privacy as being pertinent to big data applications in the insurance industry. Both Swiss and US respondents consistently ranked privacy, transparency, and equal treatment as the top three values most frequently implicated in the scenario. Notably, solidarity, which serves as a foundational ethical principle within insurance practices (Barry 2020; Barry & Charpentier 2020), exhibited disparity in the rankings of Swiss and US respondents. While solidarity ranked fourth among Swiss respondents, it placed only seventh among US respondents.

For the main study, we decided to focus on privacy and equal treatment (fairness), as they are highly ranked but quite different in content, as well as on solidarity, as it is a foundational principle within insurance practices.

**Table A1.** *Ranking of and percentage by which the values were selected by Swiss and US respondents*

|  | Ranking | | | Percentage | | |
| --- | --- | --- | --- | --- | --- | --- |
| Values | Swiss | US | Swiss | | US |  |
| Privacy | 1 | 1 | 65.1% | | 76.4% |  |
| Transparency | 2 | 2 | 49.4% | | 54.2% |  |
| Equal treatment (Fairness) | 3 | 3 | 48.3% | | 51.7% |  |
| Solidarity | 4 | 7 | 37.5% | | 1.5% |  |
| Security | 6 | 4 | 23.8% | | 34.3% |  |
| Autonomy | 5 | 5 | 26.1% | | 25.5% |  |
| Freedom | 7 | 6 | 10.0% | | 19.2% |  |

**Supplementary Information —** *The Survey Items*

**Perceived benefits of big data**

The term big data refers to very large amounts of data from different data sources (e. g., data from search queries on the Internet, from the smartphone, texts and photos from social media, credit card payments). These data can be analyzed using computer-based methods. Thus big data can identify patterns and trends and uncover relationships. At first glance, the connections do not always have to be completely logical.

Do you associate “big data” with advantages or disadvantages? (5-point scale, ranging from *many advantages* to *many disadvantages*)

**Endorsements of protected values**

*Note*: Items in bold made it into the final analyses.

**a) Equal treatment** (5-point scale, ranging from *strongly disagree* to *strongly agree*)

Equal treatment can play a role in assessing the offer of a car liability insurance that uses big data. By equal treatment, we mean: people are not treated favorably or unfavorably compared to others because of, e.g., their gender, skin color, ethnic origin, immigrant background, religion or sexual orientation. To what extent do you agree with the following statements? Equal treatment is a value ...

- **that one should not sacrifice, no matter the utility, financial or otherwise**. [PV_equal1]
- **that is non-negotiable.** [PV_equal2]
- where cost-benefit considerations are acceptable. [PV_equal3]

**b) Privacy** (5-point scale, ranging from *strongly disagree* to *strongly agree*)

When evaluating the offer of car liability insurance that uses big data, privacy can play a role. By privacy, we mean: personal data is protected so that no one gets access to details about people's lives; people have control of whom has access to personal information (e.g., private photos, posts on social media, health records). To what extent do you agree with the following statements? Privacy is a value ...

- **that one should not sacrifice, no matter the utility, financial or otherwise.** [PV_privacy1]
- **that is non-negotiable.** [PV_privacy2]
- where cost-benefit considerations are acceptable. [PV_privacy3]

**c) Solidarity** (5-point scale, ranging from *strongly disagree* to *strongly agree*)

When evaluating the offer of car liability insurance that uses big data, solidarity can play a role. By solidarity, we mean: a group (e. g., insured people, employees, society as a whole) supports group members in need. To what extent do you agree with the following statements? Solidarity is a value…

- **that one should not sacrifice, no matter the utility, financial or otherwise.** [PV_solidarity1]
- **that is non-negotiable.** [PV_solidarity2]
- where cost-benefit considerations are acceptable. [PV_solidarity3]

**Objections to big data applications**

**a) Scenario Driving style** (assessing data on customer’s driving behavior)

A car liability insurer uses data from tachographs (also called black box). The tachograph records the speed, acceleration and braking behavior of the driver. Based on the recorded data, one finds a link between data on driving style and accident risk. Result: The insurance lowers the premiums of low-risk drivers; premiums for high-risk drivers do not change.

- How acceptable do you think is this offer? (5-point scale, ranging from *not at all acceptable* to *highly acceptable*) [Obj_driving1]
- How outrageous do you think is this offer? (5-point scale, ranging from *not at all* outrageous to *highly* outrageous) [Obj_driving2]

**b) Scenario Social media** (assessing data on the number of spelling mistakes the driver makes in his or her social media profiles)

A car liability insurer uses data from social media. From the social media, data is collected on the number of spelling mistakes that a driver makes in his or her profiles. Based on the recorded data, one finds a link between the number of spelling errors and the accident risk. Result: The insurance lowers the premiums of low-risk drivers; premiums for high-risk drivers do not change.

- How acceptable do you think is this offer? (5-point scale, ranging from *not at all acceptable* to *highly acceptable*) [Obj_ socmedia1]
- How outrageous do you think is this offer? ((5-point scale, ranging from *not at all* outrageous to *highly* outrageous) [Obj_ socmedia2]

**c) Scenario Online shopping** (assessing data on the type of clothing the customer purchases through online shopping)

A car liability insurer uses data about the type of clothing that a driver buys in online clothing stores. Direct sex discrimination is banned in many countries. Based on the recorded data, one finds an indirect link between gender and accident risk. Result: The insurance lowers the premiums of low-risk drivers; premiums for high-risk drivers do not change. As a result, female drivers usually pay less than male drivers do.

- How acceptable do you think is this offer? (5-point scale, ranging from *not at all acceptable* to *highly acceptable*) [Obj_ shopping1]
- How outrageous do you think is this offer? (5-point scale, ranging from *not at all* outrageous to *highly* outrageous) [Obj_ shopping2]

**d) Scenario Creditworthiness** (assessing data on the driver’s creditworthiness)

A car insurer uses data about online shopping and e-banking to calculate the credit rating of a person. Based on the recorded data, one finds a link between credit rating and accident risk. Result: The insurance lowers the premiums of low-risk drivers; premiums for high-risk drivers do not change. As a result, wealthy people usually pay less than low-income people do.

- How acceptable do you think is this offer? (5-point scale, ranging from *not at all acceptable* to *highly acceptable*) [Obj_ credit1]
- How outrageous do you think is this offer? (5-point scale, ranging from *not at all* outrageous to *highly* outrageous) [Obj_ credit2]

**Trust**

Various institutions and private companies capture and store personal information.

To what extent do you trust the following institutions to protect your personal information? (5-point scale, ranging from *distrust strongly* to *trust strongly*)

- The government [Trust_government]
- The media [Trust_media]
- Business enterprises in general (all industries) [Trust_business]
- Insurance companies [Trust_insurance]

**Trade-off resistance**

An insurer offers you a car liability insurance, which includes the collection of the following data with your consent: Data on your driving style from a tachograph (also called black box). A tachograph records the speed, acceleration and braking behavior of the driver; data from social media that you use; data about your credit rating. The insurer uses this data to calculate the accident risk of its customers.

- Would you accept the offer? (*I would accept this offer in any case* / *I would accept this offer under certain circumstances* / *I would never accept this offer*)
